# Severity of Hepatitis C Virus (Genotype-3) Infection Positively Correlates with Circulating MicroRNA-122 in Patients Sera

**DOI:** 10.1155/2014/435476

**Published:** 2014-02-18

**Authors:** Subodh Kumar, Yogesh Kumar Chawla, Sujata Ghosh, Anuradha Chakraborti

**Affiliations:** ^1^Department of Experimental Medicine and Biotechnology, Post Graduate Institute of Medical Education and Research, Chandigarh 160012, India; ^2^Department of Hepatology, Post Graduate Institute of Medical Education and Research, Chandigarh 160012, India

## Abstract

*Introduction*. Hepatitis C virus (genotype-3) causes acute and chronic hepatitis infection predomination in India. The infectious phase of the virus requires various host factors for its survival and subsequent viral particle production. Small RNA molecules like microRNA-122 (miR-122) are one such factor mostly present in the liver and play a supportive role in viral replication. *Objective*. In this study, diagnostic potential of miR-122 is evaluated in the sera of chronic hepatitis C patients. *Methods*. miRNAs were isolated from the sera samples of patients as well as controls and miR-122 expression was quantified by real-time PCR. *Results*. A significant augmentation was observed in the level of circulating miR-122 (median level, 0.66 versus 0.29, *P* = 0.001) in patients compared to controls with ROC value of 0.929 ± 0.034 (*P* < 0.001). Interestingly, miR-122 level also depicted a significant positive correlation with serum ALT (*r* = 0.53), AST (*r* = 0.44), and viral load (*r* = 0.52). *Conclusion*. The study thus unveiled the role of miR-122 as a plausible diagnostic biomarker during HCV genotype-3 infection in India.

## 1. Introduction

Hepatitis C virus (HCV) is the leading cause of chronic hepatitis C (CHC) infection, which infects approximately 3% of the world population [1]. HCV is a positive stranded RNA virus with a genome size of 9.6 Kb that frequently replicates and survives in the liver tissue. HCV is divided into six distinct genotypes and multiple subtypes distributed throughout the world. In India, approximately 12-13 million people suffer predominantly from HCV genotype-3 infection (63.4%) followed by genotype-1 (31%) and genotype-2 (5.6%) [2, 3]. In North India, more than 76% of CHC cases were specifically associated with genotype-3 [4]. The viral infection is diagnosed by detection of anti-HCV antibodies and HCV RNA in the patient's sera. The pathogenic impact of HCV on the human liver is determined by the routine liver function test (LFTs) particularly with alanine transaminase (ALT) and aspartate transaminase (AST) level. However, an increased level of ALT and AST has been reported during muscle inflammation and in brain disorders, respectively [5]. Dufour et al. observed a normal ALT level usually in one-third of hepatitis C virus infected patients [6]. Such, uncertain behavior of these enzymes reduces their prognostic potential in case of HCV infection.

HCV survival and subsequent viral particle production need various host factors such as CD81, TGF-*β*, and CypA proteins as well as microRNAs (miRNAs), specifically microRNA-122 (miR-122) [7, 8]. The expression of these factors is altered in host cells during viral infection. miRNAs are important regulators of a wide range of physiological and pathological processes such as cellular development, metabolism, viral infection, and cancer [9–11]. miR-122 is a liver specific miRNA whose biological functions are to maintain liver homeostasis, cholesterol metabolism, and fetal liver development [12]. It also regulates HCV replication positively in cultured human hepatoma cells, which stably expresses HCV replicon by binding to the 5′UTR of the HCV genome [8]. Furthermore, inhibition of miR-122 expression by antisense oligonucleotides reduces the HCV replication and translation, yet the clinical impact of miR-122 as diagnostic or prognostic molecule is largely unknown [11, 13].

Recently, few studies have indicated that miR-122 exhibits different expression profile in the liver and the sera of hepatitis C patients [11, 14]. However, limited information is available regarding HCV genotype-3 infection and status of miR-122 in patients sera as well as its association with viral and the host parameters. Hence, in the present study, expression level of miR-122 was assessed in HCV genotype-3 infection along with other host parameters in order to explore the association of miR-122 with the disease severity.

## 2. Materials and Methods

### 2.1. Study Subjects

Blood samples were collected from chronic hepatitis C patients attending the liver clinic of PGIMER, Chandigarh, India, after receiving their informed consent. Patients positive for anti-HCV antibodies, high HCV genotype-3 viral load (>10,000 IU/mL), and without any antiviral treatment were enrolled in the study (Table 1). HCV viral load and genotype-3 were already estimated by the clinical laboratories. Blood samples were collected from healthy individual to be used as control. This study was approved by the Institute Ethical Committee (Memo no. 7969-PG-1TRG-09/17567).

### 2.2. Detection of Anti-HCV Antibody in Patient's Serum

Initially, 2 mL of blood sample was collected from each CHC patient in plain vials and serum was separated within 2 hrs of blood collection by centrifugation at 2,000 rpm for 10 min at 4°C. Serum was collected in a fresh tube and stored at −80°C till further use. HCV infection was detected by third generation SD HCV-ELISA 3.0 kit containing recombinant HCV antigens for core, NS3, NS4, and NS5 proteins (Standard Diagnostics, Inc. Korea) following manufacturer's protocol.

### 2.3. Liver Function Tests (LFT) of Patient Samples

Samples were processed for liver function test like ALT, AST, alkaline phosphatase (ALP), serum bilirubin, albumin, and globulin using specific kits (Swemed Diagnostics, Bangalore, India) according to manufacturer's instructions in fully Automated Biochemical Analyzer (Hitachi, Tokyo, Japan) (Table 1).

### 2.4. miRNA Isolation and Detection

miRNAs were isolated from the serum samples by using MagMAX viral RNA isolation kit (Ambion, USA) as per manufacturer's instructions [15]. Briefly, 400 *μ*L of serum sample was mixed with magnetic beads and separated on the magnetic stand. 20 *μ*L of elution buffer was added to elute the purified miRNA in a nuclease-free container and further detected on the 12% denaturing urea poly acrylamide gel electrophoresis (PAGE). Urea (4.8 gm) with 40% acrylamide was dissolved in 10x TBE in a final volume up to 10 mL with double distilled water. The gel was prepared by mixing above solution with 10% APS and 10 *μ*L of TEMED. miRNA samples were denatured in a water bath and run on urea PAGE at 240 Volt and 40 Amp. The gel was stained with ethidium bromide (0.5 *μ*g/mL in 1x TBE) and visualized under UV transilluminator.

### 2.5. Quantification of miR-122 Expression by Real-Time PCR

#### 2.5.1. Polyadenylation of miRNAs

Each miRNA molecule was elongated by polyadenylation reaction at 3′ end using miRNA Ist strand cDNA synthesis kit (Stratagene, USA). miRNA (1 *μ*g) was incubated at 37°C for 30 min in a reaction mixture containing 5x poly A polymerase buffer, rATP (10 mM), *E. coli* poly A polymerase, and RNase-free H_2_O. The reaction was terminated at 95°C for 5 min and immediately proceeded for Ist strand cDNA synthesis.

#### 2.5.2. cDNA Synthesis of Poly A Tailed miRNA

cDNA synthesis reaction mixtures were prepared by adding 1x RT buffer, dNTPs (100 mM), RT adaptor primer (10 *μ*M), RNase block, polyadenylated RNA, and RNase-free H_2_O. The annealing of adaptor primer to poly A tail creates a universal sequence tag followed by its incorporation at the 5′ end of cDNA. Reaction mixture was first incubated at 55°C for 5 min and then at 25°C for 15 min. Thereafter, it was transferred to 42°C and incubated for 30 min to allow reverse transcription. The reaction was terminated at 95°C for 5 min. Finally, 20 *μ*L RNase-free water was added to each reaction and placed on ice for immediate use.

#### 2.5.3. Real-Time PCR for miR-122

The expression of miR-122 was quantified by real-time PCR (qRT-PCR) analysis using high-specificity miRNA QPCR core reagent kit (Stratagene, USA) containing 10x core PCR buffer, 50 mM MgCl_2_, 20 mM dNTP mix, 20x EvaGreen dye, PCR enzyme blend, nuclease-free PCR H_2_O, universal reverse primer (3.125 *μ*M) (5′-CTCAACTGGTGTCGTGGA-3′), and miR-122 specific forward primer (5′-TGGAGTGTGACAATGGTGTTTGT-3′). The highly conserved U6 small nuclear RNA (snRNA) was taken as internal control (forward: 5′-CGCTTCGGCAGCACATATACTAA-3′ and reverse: 5′-TATGGAACGCTTCACGAATTTGC-3′) [16]. Reaction conditions were 95°C for 5 min to denature DNA templates, followed by 40 cycles of 95°C for 10 sec denaturation, 58°C for 15 sec annealing, and 72°C for 25 sec extension. The relative levels of serum miR-122 in patients versus controls were determined in terms of their fold change using the formula (2^−ΔΔCt^) [17], where ΔCt was calculated by subtracting Ct of U6 from Ct of miR-122 and ΔΔCt value was obtained by subtracting ΔCt of miR-122 in controls from the ΔCt of miR-122 in patients [10]. The qRT-PCR was performed in triplicates and data were expressed as the mean ± SD from separate experiments.

### 2.6. Statistical Analysis

Data was analyzed using the SPSS software version 17.0 (USA). The level of miR-122 in CHC patients and controls was calculated using the non-parametric Mann-whitney *U* test. The receiver operating characteristic (ROC) curve was plotted by differentiating CHC patients from healthy controls [18]. The diagnostic value of miR-122 for detection of CHC patients was determined according to the area under the ROC curve (AUC) value, the sensitivity, and the specificity. The nonparametric statistical significance for correlation was determined using Spearman's nonparametric rank test. The correlation coefficients (*r*) were calculated using Spearman correlation. *P* values < 0.05 were considered to be significant.

## 3. Results

### 3.1. Clinical Profile of Patients

The treatment naïve CHC patients and healthy controls enrolled for the study are summarized in Table 1. The average ages of CHC patient group and control groups were 38.08 ± 10.81 and 32.53 ± 9.63 years, respectively. Patients (*n* = 25) were positive for anti-HCV antibody with genotype-3 while none of the healthy volunteer carried HCV. Viral load was high in patients (>10,000 IU/mL) with a mean of 6.18 × 10^6^ (IU/mL). Similarly, mean values of ALT, AST, and globulin were higher compared to reference value.

### 3.2. miR-122 Expression in HCV Genotype-3 Infected Patient's Sera

**Figure 1:**
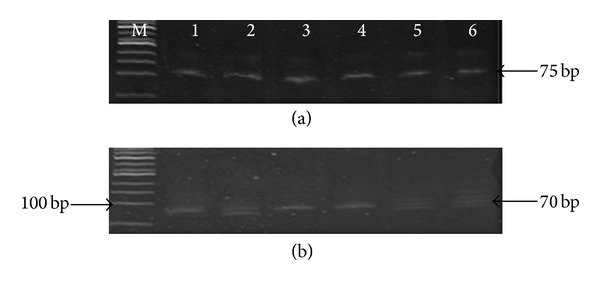
12% denaturing UREA-PAGE showing expression (Lanes 1–6) of (a) U6 snRNA (75 bp) and (b) miR-122 expression (70 bp) detected in different serum samples. *M* = 50 bp DNA ladder (Fermentas, USA).

**Figure 2:**
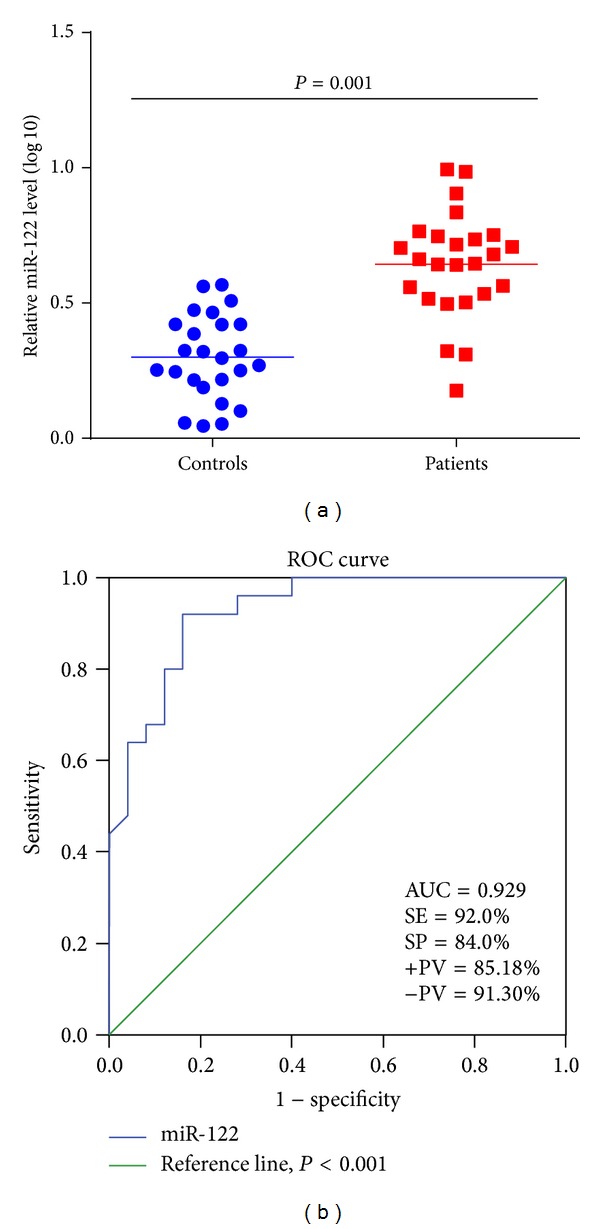
(a) Scattered plot diagram showing level of miR-122 expression in CHC patients and controls. The data are expressed as the mean ± SD from separate experiments performed in triplicates. Here, ΔCt value was normalized by U6 snRNA at log 10 scale which is shown at *y*-axis. The median value of miR-122 level is significantly higher in patients than controls, 0.66 versus 0.29 (*P* = 0.001). (b) Receiver operating characteristic (ROC) curve for miR-122 in 25 chronic hepatitis C patients. The diagnostic value of miR-122 was assessed by discriminating CHC patients from healthy controls based on the ΔCt value. SE: sensitivity, SP: specificity, +PV: positive predictive value, and −PV: negative predictive value.

miRNAs were isolated from the sera samples of CHC patients (*n* = 25) and analyzed by qRT-PCR. Serum U6 snRNA and miR-122 expression were detected on the 12% denaturing UREA-PAGE (Figures 1(a) and 1(b)). U6 snRNA was selected as internal references for quantification of serum miRNAs because it is ubiquitously expressed in patients and even in controls. miR-122 expression was quantified by subtracting the Ct value of U6 from the Ct value of miR-122 in patients and controls. The relative expression level of miR-122 was determined in both groups and the median level of miR-122 was found to be significantly high (0.66 versus 0.29, *P* = 0.001) in patients than controls (Figure 2(a)) confirming elevation of circulating miR-122 in serum of patients.

### 3.3. miR-122 as Diagnostic Marker

To evaluate the diagnostic potential of circulating miR-122 in CHC patients, the area under curve (AUC) value from ROC curve was determined in patients as compared to healthy volunteers. The ROC curve was plotted according to the ΔCt value of the miR-122 in patient's versus controls. The calculated AUC value was up to 0.929 ± 0.034 (*P* < 0.001) (Figure 2(b)) and the 95% confidence interval was 0.861 to 0.996. The cut-off value was 2.99 with a sensitivity of 92% (95% confidence interval: 73.97% to 99.02%) and specificity was 84% (95% confidence interval: 62.62% to 95.26%). Further, the positive predictive value (PPV) and negative predictive value (NPV) were 85.18% and 91.30%, respectively. Thus, ROC curve analysis confirmed miR-122 as a valuable molecule capable of discriminating the patients and healthy individuals.

### 3.4. miR-122 Expression Associated with HCV Viral Load

**Figure 3:**
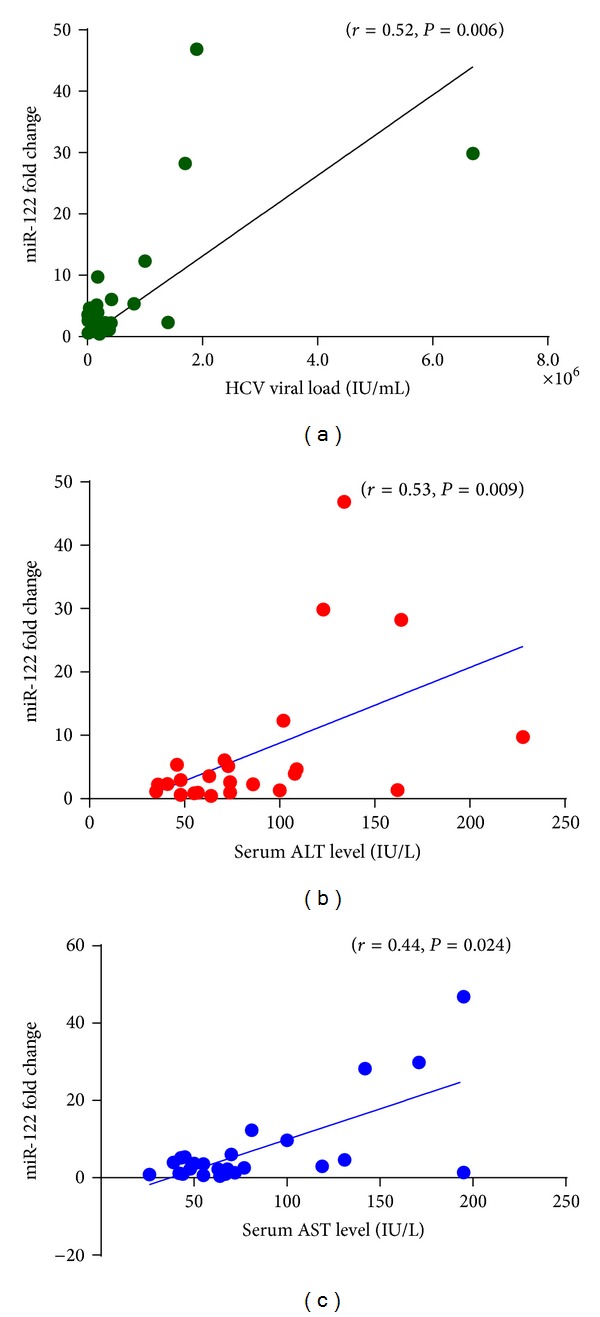
Scattered plot diagrams showing the significant positive correlation of serum miR-122 level with (a) HCV viral load, (b) serum ALT level, and (c) serum AST level.

We investigated the correlation between miR-122 expression and HCV viral load in the patient's sera. Level of miR-122 was calculated in terms of their fold change, which showed a mean value of 7.17 ± 11.25 fold higher expression in patients as compared to control group. Further, Spearman rank correlation coefficient (*r*) was determined between miR-122 level and serum HCV viral load (Figure 3(a)). Viral load of patients 6.18 ± 13.59 (10^6^) IU/mL was plotted against fold change in miR-122 which demonstrated a significant positive correlation (*r* = 0.52, 95% confidence interval: 0.15 to 0.76, *P* = 0.006). Hence, the close association of miR-122 with disease severity in terms of HCV viral load was established.

### 3.5. miR-122 Expression with Liver Function Tests

Spearman rank correlation (*r*) analysis between serum level of miR-122 and liver function parameters was also performed. Here, values of serum ALT (86.56 ± 46.90 IU/L), AST (82.48 ± 48.76 IU/L), ALP (227.48 ± 118.20 IU), serum bilirubin (0.79 ± 0.69 mg%), albumin (4.21 ± 1.02 gm%), and globulin (3.55 ± 0.57 gm%) were plotted on *x*-axis and miR-122 fold change (7.17 ± 11.25) values were plotted on *y*-axis. The level of miR-122 was correlated only with serum ALT and AST activity with significant positive correlation of (*r* = 0.53, 95% confidence interval: 0.15 to 0.77, *P* = 0.009) and (*r* = 0.44, 95% confidence interval: 0.052 to 0.72, *P* = 0.024) respectively (Figures 3(b) and 3(c)). While other parameters of liver function test did not show any significant correlation. Although serum HCV viral load showed a positive correlation with ALT and AST levels, it was insignificant. Therefore, association of miR-122 with other host parameters further confirmed its decisive role in disease assessment.

## 4. Discussion

HCV infects approximately 1.8–2.5% of Indian population accounting for 15–20% of all chronic liver disease and about 5–10% of hepatocellular carcinoma (HCC) [19, 20]. The initial diagnosis of liver diseases is carried out by routine liver function tests like serum level of ALT, AST, ALP, bilirubin, albumin, globulin, and so forth. The levels of these enzymes or proteins are altered during hepatic injuries, hepatitis B or C infection, liver cirrhosis, and HCC [6]. Usually in hepatitis C virus infection the levels of both ALT and AST are examined routinely in the patient's serum however, in some cases; the level of these enzymes remains unaltered or under normal value [21]. Even though ALT and AST are primarily localized in liver, their activity also increases heart, brain, and skeletal muscle disorders [5, 22]. The activity of ALT and AST is ambiguous as it can be correlated with hepatic and extrahepatic diseases which provoked us to search for an alternate biomarker for HCV infection.

Dennis and Chiu discovered a new approach for the clinical diagnosis of diseases by detecting the circulating nucleic acids in the peripheral blood [23]. The circulating form of mRNA and miRNA also offered another class of molecular marker in case of various cancers and pathogenic infections [24–26]. Wang et al. in toxic liver injuries reported a frequent increase of plasma level of miR-122 than that of ALT [27]. A similar observation in chronic hepatitis B patients has been reported by Zhang et al. [10]. Bihrer et al. correlated the serum level of miR-122, ALT, and necroinflammatory activity in CHC patients [14]. As liver cells contain high concentration of miR-122, it might be speculated that during liver injuries or pathogenic infection, miRNAs secretion is a strategy of cell adaptation for adverse conditions.

In North Indian population, genotype-3 HCV infection is more frequent among all HCV genotypes [4]. The clinical significance of miR-122 in HCV genotype-3 infected patients has not been discussed so far. Our study is primarily restricted to HCV genotype-3 virus infection with viral load beyond 10,000 IU/mL. Here, an elevation in the level of serum miR-122 and a significant positive association between miR-122 level and HCV RNA were observed. Although there is no such biological explanation regarding miR-122 elevation in the patients' sera, it may be speculated that upon viral infection miRNAs might be passively released from the damaged or dying cells. While other possible mechanism might be linked to the virus itself, that can also elicit the secretion of miRNAs from host cells to influence the cellular physiology for their own production [28]. These assumptions may be correlated with our observations that miR-122 level elevated as the severity of infection increases. Our results are also supported by the previous study of Morita et al. where hepatic miR-122 expression was weakly but positively correlated with serum HCV load [11]. Similarly, we also ascertained the association of miR-122 level with ALT and additionally with AST activity. However, in patients infected with other HCV genotypes (1 and 2), recently it has been reported that serum miR-122 level was correlated only with hepatic histology activity index (HAI) score and ALT level but not with HCV viral load and AST activity [29].

The study depicted the correlation of serum miR-122 level with HCV viral load, ALT, and AST activity in patient's sera. Besides, viral RNA also showed a positive correlation with ALT and AST activity; it was not significant (data not shown). While HCV viral RNA is significantly associated with miR-122 and likewise miR-122 showed a positive correlation with ALT and AST activity. These observations further enhance the diagnostic importance of miR-122. Apparently, miR-122 is quite stable and more sensitive in the serum whereas ALT and AST enzymes are comparatively less stable [30, 31]. Moreover, quantification of viral RNA is quite expensive and time consuming procedure. Hence, these findings authenticate miR-122 as a promising biomolecule to investigate HCV infection and monitor liver dysfunctions.

## Figures and Tables

**Table 1:** Patients clinical profile.

Factors	CHC patients group (*N* = 25)	Healthy control group (*N* = 25)
Sex (male/female)	19/6	16/9
Age (years)	38.08 ± 10.81	32.53 ± 9.63
Anti-HCV antibody (+/−)	25/0	0/25
HCV genotype-3 (+/−)	25/0	0/25
HCV viral load (IU/mL)	6.18 ± 13.59 (10^6^)	
ALT (IU/L)^1^	86.56 ± 46.90	
AST (IU/L)^1^	82.48 ± 48.76	
Serum bilirubin (mg%)^2^	0.79 ± 0.69	
ALP (IU)^3^	227.48 ± 118.20	
Albumin (gm%)^4^	4.21 ± 1.02	
Globulin (gm%)^5^	3.55 ± 0.57	

[Reference values: ^1^(2–40 IU/L), ^2^(0.2–0.8 mg%), ^3^(98–306 IU), ^4^(3.5–5 gm%), and ^5^(2.0–3.0 gm%)].
